# Exploring the evolving roles and clinical significance of circRNAs in head and neck squamous cell carcinoma

**DOI:** 10.7150/jca.96614

**Published:** 2024-05-30

**Authors:** Pengxiang Lei, Qingbo Guo, Jiewen Hao, Hui Liu, Yaofeng Chen, Feng Wu, Zhao He, Xiaolong Zhang, Nannan Zhang, Shuxin Wen, Wei Gao, Yongyan Wu

**Affiliations:** 1 Shanxi Key Laboratory of Otorhinolaryngology Head and Neck Cancer, Department of Otolaryngology Head & Neck Surgery, First Hospital of Shanxi Medical University, Taiyuan 030001, Shanxi, China; 2 Department of Otolaryngology Head & Neck Surgery, The Third Hospital of Shanxi Medical University (Shanxi Bethune Hospital), Taiyuan 030032, Shanxi, China; 3 Department of Hepatobiliary Surgery, Shenzhen University General Hospital & Shenzhen University Clinical Medical Academy, Shenzhen University, Shenzhen 518055, Guangdong, China; 4 Department of Otolaryngology Head & Neck Surgery, Shenzhen University General Hospital & Shenzhen University Clinical Medical Academy, Shenzhen University, Shenzhen 518055, Guangdong, China; 5 Shenzhen Research Institute, Northwest A&F University, Shenzhen 518000, Guangdong, China; 6 Department of Otolaryngology Head & Neck Surgery, Longgang Otolaryngology Hospital, Shenzhen 518172, Guangdong, China; 7 Shenzhen Institute of Otolaryngology & Key Laboratory of Otolaryngology, Longgang Otolaryngology Hospital, Shenzhen 518172, Guangdong, China; 8 Shenzhen University General Hospital & Shenzhen University Clinical Medical Academy, Shenzhen University, Shenzhen 518055, Guangdong, China

**Keywords:** Head and neck squamous cell carcinoma, Circular RNA, Biomarker, Non-coding RNA, Immune evasion, Cancer stem cell

## Abstract

Head and neck squamous cell carcinoma (HNSCC) represents the predominant malignancies in the head and neck region, and has limited therapeutic alternatives. Circular RNAs (circRNAs), a substantial category of non-coding RNA molecules, exert influential roles in human disease development and progression, employing various mechanisms such as microRNA sponging, interaction with RNA-binding proteins, and translational capabilities. Accumulating evidence highlights the differential expression of numerous circRNAs in HNSCC, and numerous dysregulated circRNAs underscore their crucial involvement in malignant advancement and resistance to treatment. This review aims to comprehensively outline the characteristics, biogenesis, and mechanisms of circRNAs, elucidating their functional significance in HNSCC. In addition, we delve into the clinical implications of circRNAs, considering their potential as biomarkers or targets for diagnosis, prognosis, and therapeutic applications in HNSCC. The discussion extends to exploring future challenges in the clinical translation of circRNAs, emphasizing the need for further research.

## Introduction

Head and neck squamous cell carcinoma (HNSCC) stands as the predominant subtype of head and neck cancer, ranking seventh among global malignant tumors, with over 800,000 new cases annually^1^, ^2^. Influenced by environmental factors, smoking, alcohol consumption, and human papillomavirus infection, the specific pathogenic mechanism of HNSCC remains unclear^3^, ^4^. Due to the absence of specific symptoms, HNSCC is easily ignored in the early stage, resulting in most patients being diagnosed in the advanced clinical stage. The inherent malignant biological characteristics marked by local recurrence, lymph node metastasis, and local invasion, also contribute to poor prognosis of HNSCC. Despite available clinical treatments, including surgery, radiotherapy, chemotherapy, and immunotherapy, which have demonstrated some efficacy in improving survival time and quality of life, the overall five-year survival rate for patients with HNSCC has not significantly increased over the past decades^5^-^7^. Hence, there is an urgent need to elucidate the mechanisms underlying HNSCC development, identifying biomarkers and molecular targets for early diagnosis and targeted therapy.

Non-coding RNAs refers to RNA molecules in the transcriptome that are not translated into proteins^8^. Circular RNA (circRNA), a predominant class of endogenous non-coding RNA molecules widely expressed in eukaryotic cells, plays roles in various physiological and pathological processes, including neurodegenerative diseases^9^, ^10^, cardiovascular diseases^11^, ^12^, metabolic diseases^13^, ^14^, and cancers^15^-^18^. The present study provides a comprehensive summary of biological functions and regulatory mechanisms of circRNAs in HNSCC, examining their potential applications and clinical translational value in diagnosis, prognosis, and targeted therapy. In addition, we anticipate future research directions by addressing key issues in circRNA research relevant to HNSCC.

## 1. Biogenesis and action mechanisms of circRNA

In 1976, Kolakofsky D^19^ made the pioneering discovery of circRNA in the Sendai virus. Since then, an increasing plethora of circRNAs have been continually unveiled across diverse species, including *Drosophila melanogaster*, mice, and humans^20^. Initially relegated as non-functional by-products of mRNA splicing errors, the perception of circRNAs has undergone a transformative shift. The evolution of high-throughput sequencing technology and bioinformatics has progressively deepened the understanding of circRNAs. In recent years, circRNAs have emerged as a burgeoning frontier in molecular biology and oncology research^21^-^23^.

### 1.1 Biological characteristics of circRNA

CircRNAs, lacking a 5' cap and 3' poly (A) tail, exhibit a structurally robust configuration that imparts high stability. This stability renders circRNAs resistant to degradation by exonucleases, resulting in an extended half-life compared to linear RNA^24^-^26^. Demonstrating a high degree of conservation across species, circRNAs exhibit spatiotemporal specificity in expression, with notable variations in types and abundance across different tissues, cells, and developmental stages^27^, ^28^.

**Figure 1:**
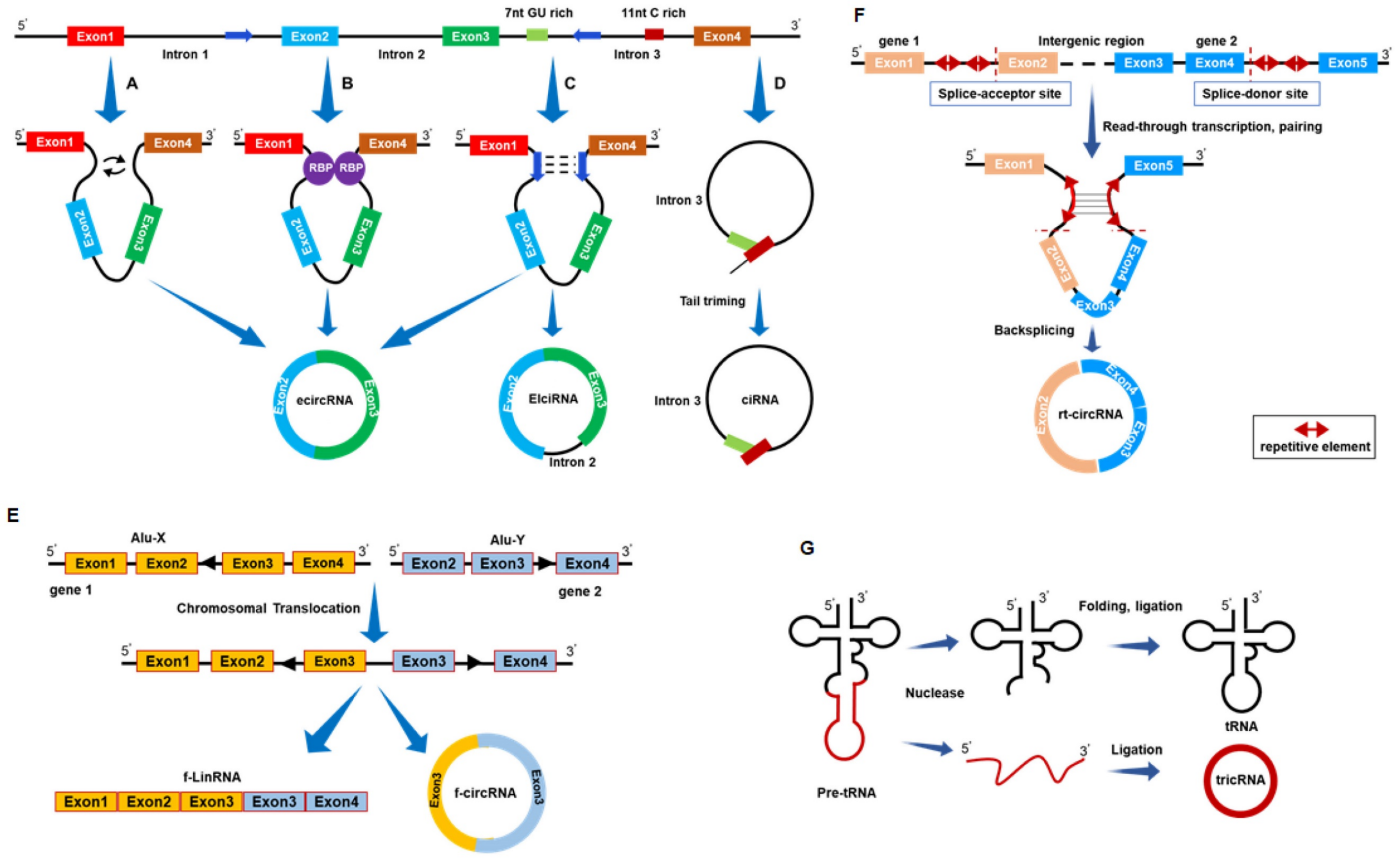
Biogenesis and classification of circRNA. (A) Lariat-driven model. (B) Intron-pairing-driven model. (C) RBP-pairing-driven model. (D) Generation of intronic circRNAs (ciRNAs). (E) Generation of fusion circRNAs (f-circRNAs). (F) Generation of read-through circRNAs (rt-circRNAs). (G) Generation of tRNA intronic circRNAs (tricRNAs). ecircRNA: exonic circRNA; EIciRNA: exonic-intronic circRNA.

The mechanisms governing circRNA formation mainly involve three models: (1) Exon skipping or Lariat-driven model; (2) RNA-binding protein (RBP)-pairing-driven model; and (3) Intron-pairing-driven model. In the Exon skipping or Lariat-driven model, precursor mRNA (pre-mRNA) undergoes partial overlapping during transcription, leading to reverse splicing of downstream 3' splice sites with upstream 5' splice sites. This process brings non-adjacent exons into proximity, forming a circular structure^29^-^32^ (Fig. 1A). In the RBP-pairing-driven model, RBPs bind to specific base sequences in flanking introns, regulating circularization through protein-protein interactions or dimer formation^33^-^35^ (Fig. 1B). In the intron-pairing-driven model, the flanking introns of downstream splice donor sites and upstream splice acceptor sites contain reverse complementary sequences, such as Alu elements. Selective splicing after base pairing leads to the formation of circRNAs with or without introns^25^ (Fig. 1C). In addition, intron circRNAs (ciRNAs) form through a 7 nt GU-rich sequence near the 5' splice site and an 11 nt C-rich sequence near the branch point, circularizing after the action of RNA polymerase II^30^ (Fig. 1D). The connection of exons from different genes on the same or different chromosomes can produce fusion circRNAs (Fig. 1E) and read-through circRNAs^36^, ^37^ (Fig. 1F). Based on origin and formation mechanism, circRNAs are classified into exonic circRNAs (ecircRNAs), exonic-intronic circRNAs (EIciRNAs), intronic circRNAs (ciRNAs), and tRNA intronic circRNAs (tricRNAs) (Fig. 1G). Among these, ciRNAs and EIciRNAs are predominantly localized in the cell nucleus, whereas ecircRNAs primarily distribute in the cytoplasm. ecircRNAs, accounting for over 80% of known circRNAs, have been the focus of extensive research^38^-^40^.

### 1.2 Action mechanisms of circRNA

#### 1.2.1 Transcription and splicing regulation

Introns containing circRNAs are predominantly located in the cell nucleus, where they interact with promoters and recruit transcriptional regulatory proteins, activating gene transcription^30^. Noteworthy examples include circACTN4, which recruits Y-box binding protein 1 to co-activate Frizzled-7 transcription^41^. Cia-MAF interacts with the MAFF promoter, recruiting the TIP60 chromatin remodeling complex to activate MAFF transcription^42^. circRap1b induces H3K14ac modification by recruiting acetyltransferase Kat7 to the Hoxa5 promoter region, resulting in Hoxa5 transcriptional activation and increased Fam3a expression^43^. circ_001659 recruits RBBP5 to the Vimentin promoter, enhancing H3K4 trimethylation at the Vimentin promoter, activating Vimentin transcription^44^.

**Figure 2:**
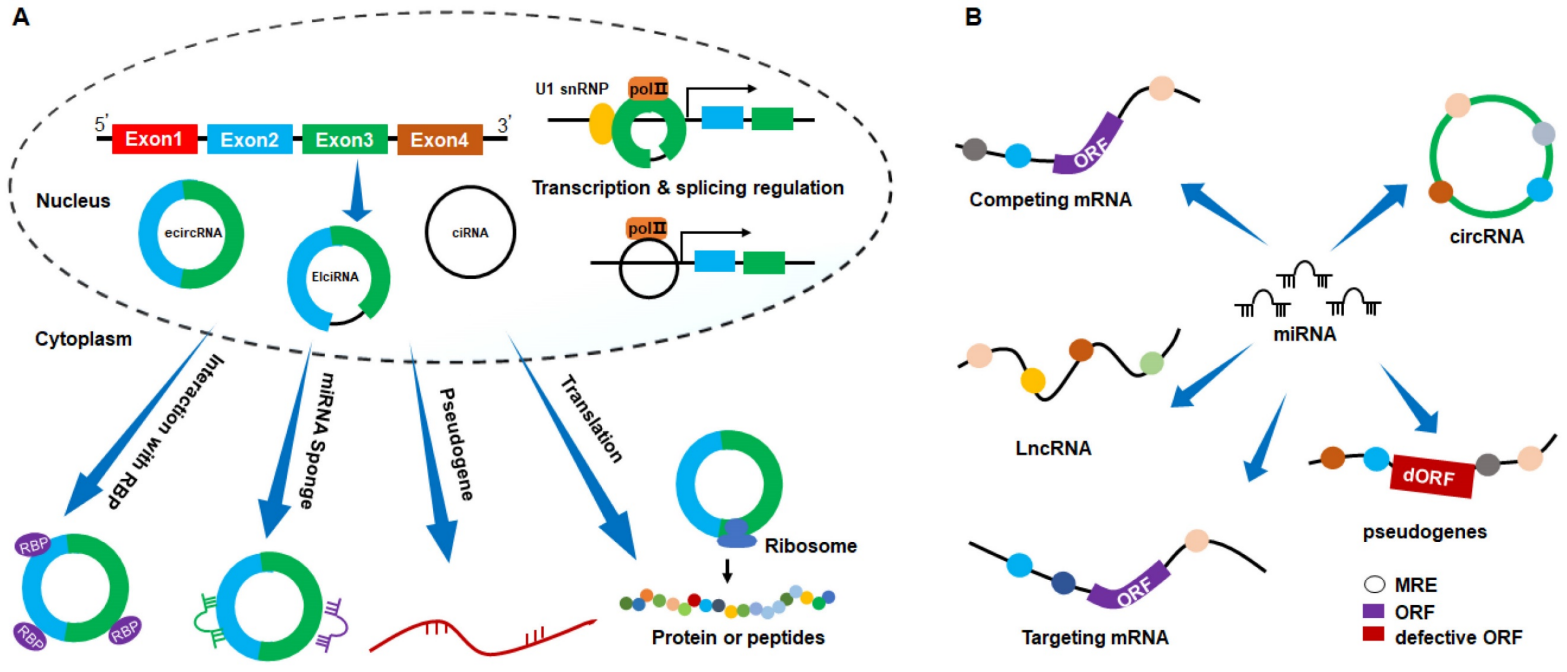
Schematic representation of the action mechanisms of circRNAs. (A) circRNAs exert biological functions through mechanisms such as miRNA sponges, RNA-binding protein interaction, transcription and splicing regulation, protein or peptide translation, and pseudogene generation. (B) miRNA binds to mRNA, LncRNA, pseudogene, and circRNA, forming a competitive binding relationship among RNA molecules that bind to the same miRNA.

Introns containing circRNAs can also interact with RNA Polymerase II, exerting regulatory effects on their parent coding genes^45^. circEIF3J and circPAIP2 form an EIciRNA-U1 SnRNP complex, binding to the U1 binding site in EIciRNA and interacting with U1 snRNA, regulating parent gene transcription by interacting with the RNA Polymerase II promoter site^46^. In addition, Xu *et al.* reported that circSMARCA5 directly binds to its parent gene site, forming an R-loop that terminates exon 15 transcription of SMARCA5^47^. Moreover, introns containing circRNAs regulate alternative splicing by influencing splicing factors. For example, circSMARCA5 modulates the pre-mRNA splicing process of VEGFA by recruiting the splicing factor SRSF1, reducing the production of VEGFA splice isoforms^48^. These studies underscore the regulatory role of circRNAs at both transcriptional and splicing levels (Fig. 2A).

#### 1.2.2 Protein or peptide translation

Traditionally, eukaryotic mRNA translation relies on the 5' cap structure. Due to the absence of a 5' cap and 3' poly (A) tail, circRNAs have been categorized as non-coding RNAs. However, recent studies have unveiled a subset of circRNAs capable of encoding proteins or peptides, serving as templates for ribosomal translation^49^, ^50^. These circRNAs feature an internal ribosome entry site-driven open reading frame, facilitating direct ribosomal recruitment and translation initiation (Fig. 2A). For instance, circ-EIF6 encodes the novel peptide EIF6-224aa, EIF6-224aa directly interacted with the oncogenic protein MYH9 to decrease its degradation by inhibiting the ubiquitin-proteasome pathway, thereby promoting proliferation and metastasis in triple-negative breast cancer^51^; circDIDO1 encodes the protein DIDO1-529, and DIDO1-529 interacted with poly ADP-ribose polymerase 1 (PARP1) and inhibited its activity. Knockdown of circDIDO1 promoted gastric cancer cell proliferation, migration and invasion^52^; circMAPK14 functioned as a tumor suppressor by encoding a peptide of 175 amino acids (circMAPK14-175aa), which blocked the malignant progression and metastasis of colorectal cancer^53^; circAXIN1 encodes the protein AXIN1-295aa, which competitively interacts with APC to activate the Wnt signaling pathway, functioning as an oncogenic protein in gastric cancer^54^. Notably, recent studies found that RNA m6A modification enhances the initiation of circRNA protein translation^55^. In this context, circARHGAP35 undergoes m6A-dependent translation, producing an oncogenic protein^56^. Furthermore, m6A modification drives the translation of circMAP3K4 into the peptide circMAP3K4-455aa^57^.

#### 1.2.3 Interaction with RNA-binding proteins

Specific circRNAs harbor binding sites for RNA-binding proteins, enabling direct interactions^58^, ^59^ (Fig. 2A). For instance, circDLC1 binds to the RNA-binding protein HuR. This interaction impedes the binding of HuR and MMP1 mRNA, resulting in the inhibition of MMP1 expression. Consequently, it suppresses the proliferation and metastasis of hepatocellular carcinoma^60^. circCwc27 interacts with the RNA-binding protein Pur-α. This interaction inhibits Pur-α activity, playing a role in Alzheimer's disease onset and development^61^. circSETD2 interacts with HuR, diminishing the stability of YAP1 mRNA, ultimately inhibiting the progression of breast cancer^62^. Recently, Ju *et al.* identified an intron containing circRNA in HNSCC, named as circGNG7. Mechanistically, circGNG7 binds to serine residues 78 and 82 of the functional heat shock protein 27 (HSP27), hindering its phosphorylation, which reduced HSP27-JNK/P38 mitogen-activated protein kinase (MAPK) oncogenic signaling^63^.

#### 1.2.4 circRNA-derived pseudogene

Pseudogenes are genomic DNA sequences closely resembling coding genes but have lost their normal function due to the absence of functional promoters or other regulatory elements, often remaining transcriptionally inert^64^, ^65^. Studies indicate that pseudogenes originating from linear mRNAs can undergo reverse transcription and integrate into the host genome. Similarly, circRNAs can also undergo reverse transcription transposition, resulting in pseudogenes derived from processed circRNAs being inserted into the host genome, thereby altering genomic DNA composition^66^, ^67^ (Fig. 2A). To date, the functions and mechanisms of pseudogenes derived from circRNAs remain unclear.

#### 1.2.5 miRNA (microRNA) sponge

miRNAs, approximately 19-24 nucleotides long, are small endogenous non-coding single-stranded RNAs that regulate translation or induce mRNA degradation by binding to the 3'UTRs of target mRNAs. This binding is mediated by miRNA response elements (MREs) on various RNAs, including lncRNAs, pseudogenes, and circRNAs. The same miRNA can bind to multiple types of RNAs, and the competitive binding of RNAs with the same MREs to miRNAs is known as the competing endogenous RNA mechanism^48^, ^68^ (Fig. 2B). Within this mechanism, circRNA are referred to as miRNA “sponge” due to their specific adsorption of miRNAs, thus modulating the expression of downstream target genes. Numerous studies confirm the ability of circRNAs to reduce miRNA inhibitory effects on target genes, indirectly regulating target gene expression^69^, ^70^. Typically, a single circRNA harbors multiple binding sites for different miRNAs or multiple sites for the same miRNA. For instance, circTMEM59 inhibits colorectal cancer cell migration by adsorbing miR-668-3p and miR-410-3p. It also serves as a sponge for miR-147b, impeding the progression of pancreatic ductal adenocarcinoma^71^-^73^. Moreover, the same miRNA can be adsorbed by different circRNAs. For example, circKIF4A and circ_0058063 contain miR-335-5p binding sites, thereby regulating miR-335-5p target gene expression^74^, ^75^. To date, miRNA sponge is the most extensively studied mechanism of circRNA.

In summary, circRNAs exert their biological functions through various mechanisms, including transcription and splicing, interaction with RNA binding protein, translation of proteins or peptides, generation of pseudogenes, and acting as miRNA sponge.

## 2. Functional roles and mechanisms of circRNA in HNSCC

### 2.1 Regulation of proliferation

circRNAs exert a pivotal role in modulating the proliferation of HNSCCs. Notably, several circRNAs, including circ_0000045, circ_0000052, circ_0023028, circ_0032822, circZNF609, circPVT1, circHIPK2, and circ-CCND1, are upregulated in both HNSCC tissues and cells, actively promoting HNSCC cell proliferation^76^-^83^. Conversely, certain circRNAs function as tumor suppressor genes, exerting inhibitory effects on HNSCC cell proliferation. For example, circ_0036722 regulates the expression of the parental gene RHCG by sequestering miR-1248, suppressing laryngeal squamous cell carcinoma (LSCC) cell proliferation^84^. circ_0000140 inhibits the proliferation of oral squamous cell carcinoma (OSCC) cells^85^. Moreover, overexpression of circRNF13 exhibits inhibitory effects on nasopharyngeal carcinoma (NPC) cell proliferation^86^.

### 2.2 Regulation of cell cycle transition

Cell cycle dysregulation is a hallmark of cancer cells, with cyclin D1 serving as a key regulator in the G1/S phase transition and playing a crucial role in cancer cell proliferation^87^, ^88^. Knockdown of circMYLK in LSCC cells results in reduced cyclin D1 expression levels, suggesting that circMYLK potentially promotes tumor cell proliferation by accelerating the cell cycle process^89^. Another study demonstrated that circPTK2 promotes cell cycle progression in LSCC cells. Knockdown of circPTK2 leads to reduced expression levels of cell cycle-related proteins, including cyclin A1, cyclin B1, and cyclin D1^90^.

### 2.3 Regulation of invasion and migration

Invasion and migration are pivotal features of malignant tumors, contributing significantly to the mortality of patients with cancer. Epithelial-mesenchymal transition (EMT) is crucial in tumor cell dissemination, orchestrating the shift from an epithelial to a more invasive, migratory mesenchymal phenotype^91^. Ma *et al.* discovered that circRNA_ACAP2 regulates the EMT process through the miR-21-5p/STAT3 axis, inhibiting HNSCC cell migration^92^. circ_0000140 binds to miR-31, upregulating the target gene LATS2 expression, thereby inhibiting the EMT process in OSCC cells^85^. Furthermore, Pei *et al.* identified that circFOXM1 upregulates Smad2 gene expression by sequestering miR-136-5p, promoting the EMT process in NPC cells^93^. Liu *et al.* demonstrated that EBV-encoded circRPMS1 fosters the EMT in NPC cells by sequestering multiple miRNAs, including miR-203, miR-31, and miR-451^94^.

### 2.4 Regulation of angiogenesis

The growth and metastasis of cancer cells rely on tumor angiogenesis, a process facilitated by the collective action of cancer cells, stromal cells, and their secretions. Given that VEGF is pivotal in promoting cancer cell growth through angiogenesis, circRNAs exert influence by directly or indirectly modulating VEGF expression levels^95^, ^96^. Gong *et al.* discovered that circBFAR promotes ki-67, MMP2, and VEGFA protein expression by binding to miR-31-5p, facilitating the generation of new blood vessels in LSCC^97^. In LSCC, silencing circSHKBP1 leads to a significant reduction in MMP2 and VEGFA expression, resulting in the inhibition of LSCC cell invasion and angiogenesis^98^. Silencing circ-ZNF609 in NPC results in decreased VEGF expression levels, along with a noticeable downregulation of VEGFR1 and VEGFR2 protein expression, suggesting that circ-ZNF609 may play a role in promoting angiogenesis in NPC^99^.

### 2.5 Regulation of immune evasion

Immune surveillance mechanisms play a pivotal role in identifying and eliminating cancer cells. Central to this process is PD-1 (Programmed death-1), a critical immune checkpoint molecule primarily expressed in immune cells. Its interaction with the ligand PD-L1 (programmed death-ligand 1) on cancer cells prevents the activation of tumor antigen-specific T cells, contributing to the immune evasion of cancer cells^100^, ^101^. circ_0000052 upregulates PD-L1 expression by sequestering miR-382-3p, thereby promoting the malignant progression of HNSCC^77^. Ge *et al.* demonstrated that EBV-encoded circBART2.2 expression promotes PD-L1 transcription through the binding of circBART2.2 to the helicase domain of RIG-I and the activation of transcription factors IRF3 and NF-κB, resulting in immune evasion of NPC^102^.

### 2.6 Regulation of apoptosis

circRNAs have a significant influence in regulating apoptosis in HNSCC through the modulation of pro-apoptotic and anti-apoptotic genes within apoptotic signaling pathways. Knockdown of circ_0044520 upregulates Bax (BCL2 Associated X) expression and simultaneously reduces BCL2 (B-cell lymphoma 2) expression, promoting apoptosis of LSCC cells^103^. Knockdown of circ_0000285 enhances Caspase-3 activity, upregulates Bax protein levels, and downregulates BCL2 protein levels. These alterations indicate the ability of circ_0000285 to inhibit apoptosis in NPC cells^104^. Silencing circRNA_100290 increases Caspase-9 expression in LSCC cells, suggesting that circRNA_100290 suppresses apoptosis^105^. In addition, hsa_circ_0002162 exhibits increased expression in Tongue squamous cell carcinoma. Silencing hsa_circ_0002162 leads to an increase in apoptotic protein Caspase-3 expression^106^.

### 2.7 Regulation of autophagy

Autophagy, a cellular process crucial for maintaining homeostasis, involves the engulfment and digestion of damaged or aging proteins and organelles by lysosomal hydrolases. P62 and LC3 serve as markers reflecting autophagic activity. In conditions of low autophagy or inhibition, P62 accumulates in the cytoplasm, while the LC3-II/I ratio indicates the level of autophagy^107^-^110^. Studies have shown that autophagy can exert dual effects on tumor occurrence and progression^111^, ^112^. Overexpression of circ-PKD2 in OSCC cells results in an increased LC3-II/I ratio and decreased P62 levels, suggesting that circ-PKD2 promotes autophagy in OSCC cells^113^. Conversely, overexpression of circPARD3 leads to decreased LC3-II levels and increased P62 levels in LSCC cells, indicating that circPARD3 inhibits autophagy in LSCC^114^.

### 2.8 Regulation of chemoradiotherapy sensitivity

Chemotherapy and radiotherapy are pivotal in cancer treatment, yet resistance poses a significant challenge, impacting treatment efficacy and contributing to poor prognosis in patients with HNSCC. circRNAs participate in the regulation of chemoradiotherapy sensitivity in cancer cells^115^, ^116^. circCUX1, upregulated in radiotherapy-resistant hypopharyngeal squamous cell carcinomas (HSCC) tissues, is implicated in promoting resistance. Knockdown of circCUX1 enhances the release of inflammatory cytokines IL-1β and IL-18, thereby augmenting the sensitivity of HSCC to radiotherapy^117^. circATRNL1 enhances OSCC sensitivity to radiation by promoting target gene PTEN expression, which is achieved through the sequestration of miR-23a-3p^118^. circ-PKD2 promotes the sensitivity of OSCC to cisplatin both *in vitro* and *in vivo*. Its mechanism involves inhibiting miR-646 and promoting Atg13-mediated autophagy^113^. Knockdown of circNRIP1 increases the sensitivity of NPC cells to 5-Fu and CDDP *in vitro*^119^. circCRIM1 competitively binds to miR-422a, counteracting its inhibitory effect on FOXQ1 and promoting resistance of NPC cells to docetaxel^120^.

### 2.9 Regulation of stem cell properties

Cancer stem cells, with their ability for self-renewal and differentiation into diverse cancer cell types, underlie the malignancy of cancer, contributing to recurrence, metastasis, and chemoradiotherapy resistance^121^. In HNSCC, cancer stem cells are increasingly recognized as pivotal players in its pathogenesis. Chen *et al.* showed that the knockdown of circSHKBP1 inhibits stem-like properties in LSCC and suppresses tumor growth. This regulatory role is attributed to the ability of circSHKBP1 to sequester miR-766-5p, consequently enhancing HMGA2 expression and promoting LSCC progression^98^. circFAT1 promotes cancer stem cell characteristics by activating STAT3. Knockdown of circFAT1 reduces HNSCC cell sphere formation *in vitro*^122^.

**Figure 3:**
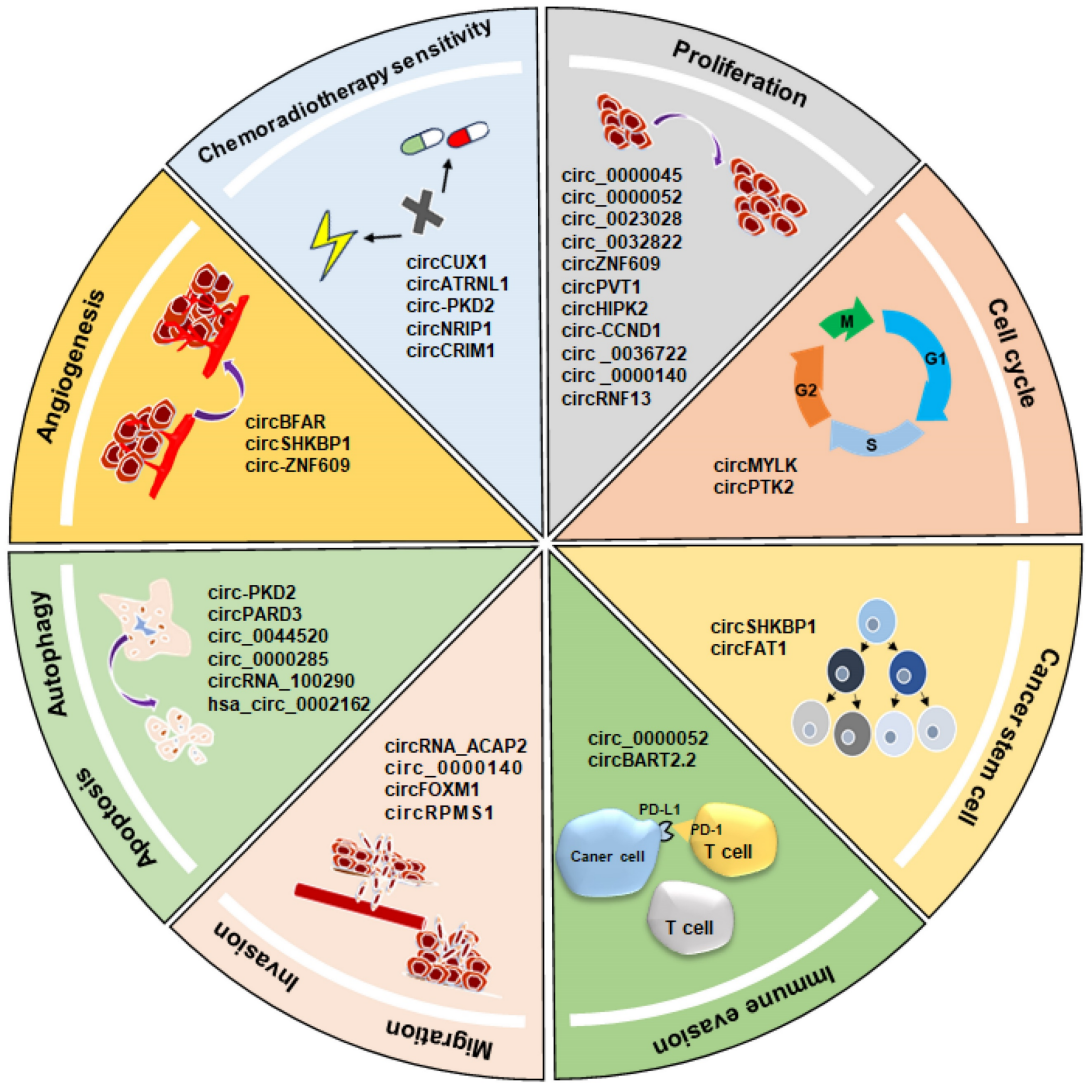
Regulatory role of circRNAs in HNSCC, including cell proliferation, cell cycle, invasion, migration, angiogenesis, immune evasion, apoptosis, autophagy, and cancer stem cell maintenance.

In summary, circRNAs function as either oncogenes or tumor suppressor genes, modulating HNSCC-related key signaling pathways. Their regulatory influence extends across various aspects, including cell proliferation, cell cycle transition, migration, invasion, angiogenesis, apoptosis, autophagy, and cancer stem cell maintenance. This comprehensive regulatory role underscores their significance in shaping the growth, recurrence, metastasis, and chemoradiotherapy sensitivity in HNSCC (Fig. 3).

## 3. Potential of circRNA as diagnostic and prognostic biomarkers in HNSCC

The identification of specific biomarkers for HNSCC is crucial for non-invasive diagnostics and accurate prognosis assessment. circRNAs, characterized by high stability, diverse types, and spatiotemporal specificity, present unique advantages as potential biomarkers in HNSCC due to their presence in various bodily fluids. Increasing evidence suggests that circRNAs have significant potential in HNSCC diagnosis and prognosis, potentially evolving into early screening and prognostic markers for patients with HNSCC^123^-^125^. hsa_circ_0003829 exhibits significantly lower expression in OSCC tissues compared to adjacent normal tissues, and the expression level of hsa_circ_0003829 was correlated with lymph node metastasis and clinical staging. Receiver operating characteristic curve analysis yields an area under the curve (AUC) of 0.81, sensitivity of 70%, and specificity of 80%, suggesting that hsa_circ_0003829 may serve as a potential diagnostic marker for OSCC^126^. circRNA_103862 upregulates in LSCC tissues and is closely linked to clinical staging and lymph node metastasis. It demonstrates an AUC of 0.805, with a sensitivity of 0.823 and a specificity of 0.694^127^. Furthermore, circ0019201, circ0011773, and circ0122790 upregulated in the plasma of patients with LSCC, with AUC of 0.766, 0.864, and 0.908, respectively, suggesting their potential as predictive biomarkers for LSCC^128^. Moreover, circMORC3 downregulation in HSCC tissues and plasma, with an AUC of 0.767, suggests its potential as an early diagnostic biomarker for HNSCC^129^ (Table 1).

## 4. Potential of circRNAs as molecular target for HNSCC treatment

The pivotal regulatory role of circRNAs in governing various aspects of HNSCC, including cell proliferation, invasion, migration, apoptosis, glucose metabolism, underscores their potential as molecular targets for HNSCC treatment^130^-^137^. Notably, circMTCL1 was upregulated in LSCC tissues. *In vivo* and *in vitro* experiments showed that circMTCL1 promotes the proliferation, invasion, and migration of LSCC cells, suggesting it serves as a potential therapeutic target for LSCC^138^ (Table 2).

## 5. Conclusions and perspectives

A growing body of evidence highlights significant dysregulation of circRNAs in HNSCC, with both *in vitro* and *in vivo* studies illustrating their regulatory effects on downstream target genes and signaling pathways. These circRNAs play crucial roles in governing processes such as cell proliferation, invasion, metastasis, apoptosis, and autophagy, influencing the occurrence, development, and sensitivity to chemoradiotherapy in HNSCC. Moreover, several circRNAs exhibit a significant association with clinical features and prognosis, showcasing their potential as promising biomarkers and therapeutic targets for HNSCC diagnosis, prognosis, and targeted therapy.

However, as research on circRNAs in HNSCC expands, several challenges and future research directions become apparent: (1) While circRNAs exhibit multiple mechanisms of action, current studies primarily focus on their role as miRNA sponges. The broader impact of circRNAs on transcription, splicing, protein interactions, and encoding proteins or peptides in HNSCC remains understudied. (2) The upstream regulation of circRNA expression dysregulation in HNSCC, including variable splicing and post-transcriptional modification, requires further investigation. (3) Understanding the regulatory role of circRNAs in HNSCC stem cells, considered the root of malignant behaviors and treatment resistance, offers potential insights for clinical diagnosis and treatment. (4) Compared with 2D cell models and animal models, the application of organoids and organ-on-a-chip technology presents an exciting avenue for studying the spatial structure and tissue analog of circRNAs in HNSCC, offering potential clinical transformation insights. (5) Current studies on circRNA biomarkers often feature small sample sizes. Large-scale, multi-center clinical samples are needed to validate the utility of circRNAs as biomarkers for early diagnosis and prognosis assessment. (6) Addressing the urgent challenge of altering circRNA expression levels in target cells is essential for circRNA transformation research. In conclusion, as circRNA research deepens, it holds substantial promise for clinical diagnosis and treatment of HNSCC in the future.

## Figures and Tables

**Table 1:** Potential circRNA biomarker for diagnosis and prognosis of HNSCC

circRNAs	Expression	Cancer type	Function	Clinical relevance	Reference
hsa_circ_0023305	up	LSCC	Promotes proliferation, invasion, migration	Clinical stage, lymph node metastasis	123
hsa_circ_0066755	up	NPC	Promotes proliferation, invasion, migration	Clinical stage	124
hsa_circ_0028007	up	NPC	Promotes migration, and invasion	Aggressive infiltration, and metastatic lymph nodes	125
hsa_circ_0003829	down	OSCC	-	Lymphatic metastasis, TNM stage	126
circRNA_103862	up	LSCC	Promotes proliferation, migration, invasion	Survival time	127
circ_0019201, circ_0011773, circ_0122790	up	LSCC	-	High diagnostic ability for single circRNA and combined	128
circMORC3	down	HSCC	-	T stage, tumor size	129

**Table 2:** circRNA serves as potential therapeutic target in HNSCC

circRNAs	Expression	Cancer type	Target genes	Function	Reference
circDHTKD1	up	OSCC	miR-326/GAB1	Promotes cell growth and migration, inhibits apoptosis	130
circ_0008068	up	OSCC	miR-153-3p/AGK	Promotes proliferation, migration, invasion, tube formation, glycolysis, inhibits apoptosis	131
hsa_circ_0042666	down	LSCC	miR-223/TGFBR3	Inhibits proliferation and invasion	132
circFLNA	up	LSCC	miR-486-3p/FLNA	Promotes migration	133
circ_0000215	up	NPC	miR-512-5p/PIK3R1	Promotes proliferation, migration	134
circRNA CDR1as	up	NPC	miR-7-5p/E2F3	Promotes proliferation, glucose metabolism	135
hsa_circ_0046263	up	NPC	miR-133a-5p/IGFBP3	Promotes proliferation, invasion, migration	136
circSOX9	up	NPC	miR-485-3p/SOX9	Promotes invasion and proliferation	137
circMTCL1	up	LSCC	C1QBP/β-catenin	Promotes proliferation, invasion, migration	138
